# Supplementary material for: A combination of oxygenation and driving pressure can provide valuable information in predicting the risk of mortality in ARDS patients
Source: PLoS One. 2023 Dec 13;18(12):e0295261. doi: 10.1371/journal.pone.0295261 (PMC10718417; doi:10.1371/journal.pone.0295261)
Supplement: S1 Table — (DOCX) [file pone.0295261.s001.docx]

**S1 Table. Characteristics of ARDS patients categorized by severity^a^**

| **Characteristics** | **All**  **(N=370)** | **Mild**  **(N=103, 27.8%)** | **Moderate**  **(N=196, 53%)** | **Severe**  **(N=71, 19.2%)** | ***P* Value ^b^** |
| --- | --- | --- | --- | --- | --- |
| Age (years) | 67.7 ± 16.9 | 68.3 ± 17.1 | 67.5 ± 17.2 | 67.3 ± 16.0 | 0.916 |
| Male, No. (%) | 252 (68.1%) | 73 (70.9%) | 130 (66.3%) | 49 (69.0%) | 0.713 |
| BMI (kg/m^2^) | 23.7 ± 4.9 | 22.9 ± 4.9 | 23.6 ± 4.7 | 25.1 ± 5.2 | 0.018 |
| **Admission source,** **No. (%)** |  |  |  |  |  |
| Emergency Room | 303 (81.9%) | 85 (82.5%) | 157 (80.1%) | 61 (85.9%) | 0.860 |
| Non- Emergency Room | 67 (18.1%) | 18 (17.5%) | 39 (19.9%) | 10 (14.1%) |  |
| **ICU type,** **No. (%)** |  |  |  |  |  |
| Medical | 295 (79.7%) | 81 (78.6%) | 152 (77.6%) | 62 (87.3%) | 0.203 |
| Surgical | 75 (20.3%) | 22 (21.4%) | 44 (22.4%) | 9 (12.7%) |  |
| **Severity scores** |  |  |  |  |  |
| APACHE II score | 28.0 ± 6.9 | 25.9 ± 6.3 | 27.9 ± 6.9 | 31.7 ± 5.9 | <0.0001 |
| SOFA score, Day 1 | 10.3 ± 3.5 | 9.5 ± 3.2 | 10.1 ± 3.5 | 12.1 ± 3.5 | <0.0001 |
| **Co-morbidities, No. (%)** |  |  |  |  |  |
| Cardiovascular disease | 102 (27.6%) | 35 (34.0%) | 55 (28.1%) | 12 (16.9%) | 0.130 |
| Cerebrovascular disease | 75 (20.3%) | 21 (20.4%) | 40 (20.4%) | 14 (19.7%) | 0.946 |
| Dementia | 26 (7.0%) | 10 (9.7%) | 16 (8.2%) | 0 (0%) | 0.046 |
| Chronic pulmonary disease | 76 (20.5%) | 28 (27.2%) | 38 (19.4%) | 10 (14.1%) | 0.215 |
| Rheumatic disease | 30 (8.1%) | 7 (6.8%) | 18 (9.2%) | 5 (7.0%) | 0.680 |
| Hepatic disease | 67 (18.1%) | 23 (22.3%) | 30 (15.3%) | 14 (19.7%) | 0.357 |
| Diabetes mellitus | 145 (39.2%) | 39 (37.9%) | 77 (39.3%) | 29 (40.8%) | 0.559 |
| Renal disease | 127 (34.3%) | 38 (36.9%) | 66 (33.7%) | 23 (32.4%) | 0.979 |
| Malignancy | 141 (38.1%) | 45 (43.7%) | 72 (36.7%) | 24 (33.8%) | 0.716 |
| Charlson Co-morbidity Index (CCI) | 3.6 ± 2.9 | 4.0 ± 3.0 | 3.4 ± 2.9 | 3.9 ± 3.0 | 0.203 |
| **Etiology of ARDS,** **No. (%)** |  |  |  |  |  |
| **Pulmonary** | 298 (80.5%) | 82 (79.6%) | 156 (79.6%) | 60 (84.5%) |  |
| Pneumonia | 255 (68.9%) | 70 (68.0%) | 132 (67.3%) | 53 (74.6%) | 0.507 |
| Aspiration | 37 (10.0%) | 12 (11.7%) | 19 (9.7%) | 6 (8.5%) | 0.770 |
| Pulmonary contusion | 6 (1.6%) | 0 (0%) | 5 (2.6%) | 1 (1.4%) | 0.249 |
| **Extra-pulmonary** | 72 (19.5%) | 21 (20.4%) | 40 (20.4%) | 11 (15.5%) | 0.596 |
| Sepsis (non-pulmonary) | 48 (13.0%) | 15 (14.6%) | 26 (13.3%) | 7 (9.9%) | 0.652 |
| Trauma or hemorrhagic shock | 4 (1.1%) | 2 (1.9%) | 2 (1.0%) | 0 (0%) | 0.473 |
| Pancreatitis | 4 (1.1%) | 0 (0%) | 4 (2.0%) | 0 (0%) | 0.166 |
| TRALI | 10 (2.7%) | 2 (1.9%) | 5 (2.6%) | 3 (4.2%) | 0.647 |
| **Clinical outcome** |  |  |  |  |  |
| ICU length of stay | 13.0 (8-22) | 14.0 (8-22) | 13.0 (8-21) | 12.0 (7-26) | 0.951 |
| Hospital length of stay | 29.5 (18-46) | 32 (19-48) | 30.0 (18-45) | 26.0 (14-46) | 0.273 |
| Ventilator-day | 13.0 (7-24) | 13 (7-23) | 13 (7-24) | 11 (6-26) | 0.904 |
| Hospital Mortality, No. (%) | 156 (42.2%) | 37 (35.9%) | 86 (43.9%) | 33 (46.5%) | 0.298 |

Abbreviations: ARDS, acute respiratory distress syndrome; BMI, Body Mass Index*;* ICU, intensive care unit; APCHE II, Acute Physiology And Chronic Health Evaluation II; SOFA, The sequential organ failure assessment score; CCI, Charlson co-morbidity index; TRALI, transfusion-related acute lung injury; sd , standard deviation; IQR (interquartile range).

^a^ ARDS severity determined from worst partial pressure of oxygen to fraction of inspired oxygen ratio within first 24 hours following ARDS diagnosis.

^b^ *P* value represents comparisons across the ARDS severity categories for each variable
