# Supplementary material for: A combination of oxygenation and driving pressure can provide valuable information in predicting the risk of mortality in ARDS patients
Source: PLoS One. 2023 Dec 13;18(12):e0295261. doi: 10.1371/journal.pone.0295261 (PMC10718417; doi:10.1371/journal.pone.0295261)
Supplement: S2 Table — (DOCX) [file pone.0295261.s002.docx]

**S2 Table. Mechanical ventilation and arterial blood gas on admission by ARDS severity^a^**

| Variables | All (N=370) | Mild (N=103) | | Moderate (N=196) | | Severe (N=71) | *P* Value ^b^ |
| --- | --- | --- | --- | --- | --- | --- | --- |
| **Ventilator settings, first day of ARDS (Day0)** | | | | | | | |
| Mode, No. (%) |  |  | |  | |  |  |
| Volume target, No. (%) | 305 (82.4%) | 85 (82.5%) | | 158 (80.6%) | | 62 (87.3%) | 0.602 |
| Pressure target, No. (%) | 65 (17.6%) | 18 (17.5%) | | 38 (19.4%) | | 9 (12.7%) |  |
| FiO_2_ | 0.6 ± 0.2 | 0.5 ± 0.2 | | 0.6 ± 0.2 | | 0.9 ± 0.2 | <0.0001 |
| PaO_2_ (mmHg) | 103.9 ± 53.3 | 149.4 ± 75.0 | | 90.7 ± 26.6 | | 74.6 ± 20.5 | <0.0001 |
| P/F ratio | 173.5 ± 88.4 | 276.4 ± 93.3 | | 151.4 ± 32.7 | | 85.1 ± 25.1 | <0.0001 |
| PEEP (cmH_2_o) | 8.8 ± 4.1 | 7.1 ± 2.7 | | 9.2 ± 4.7 | | 10.2 ± 3.0 | <0.0001 |
| Tidal Volume (vt/cc/kg) | 7.5 ± 1.6 | 7.9 ± 1.3 | | 7.4 ± 1.6 | | 7.0 ± 1.7 | 0.001 |
| RR (1breath/min ) | 22.2 ± 5.4 | 20.5 ± 7.8 | | 22.2 ± 5.4 | | 24.4 ± 5.4 | <0.0001 |
| PIP (mmHg) | 24.9 ± 5.9 | 23.5 ± 5.0 | | 25.0 ± 5.1 | | 26.6 ± 6.0 | 0.001 |
| P_plat_ (cmH_2_o) | 21.5 ± 5.2 | 19.5 ± 4.2 | | 21.6 ± 5.3 | | 24.2 ± 5.1 | <0.0001 |
| Driving pressure (cmH_2_o) | 12.9 ± 4.1 | 12.6 ± 4.3 | | 12.7 ± 3.8 | | 13.9 ± 4.6 | 0.1 |
| Compliance (cmH_2_o) | 32.9 ± 12.3 | 35.7 ± 13.0 | | 32.7 ± 11.9 | | 29.5 ± 11.5 | 0.008 |
| **Artery blood gas(ABG)-patient in ICU 24hrs** | | | | | | | |
| pH | 7.4 ± 0.1 | 7.38 ± 0.1 | | 7.36 ± 0.1 | | 7.33 ± 0.1 | 0.023 |
| PaO_2_ (mmHg) | 122.2 ± 98.0 | 170.8 ± 155.1 | | 106.7±51.9 | | 94.0 ± 54.3 | <0.0001 |
| PaCO_2_ (mmHg) | 41.8 ± 28.2 | 37.7 ± 10.2 | | 42.8 ± 32.9 | | 41.6 ± 13.4 | 0.179 |
| HCO_3,_ | 21.9 ± 5.0 | 21.2 ± 4.8 | | 22.4 ± 6.2 | | 21.5 ± 5.3 | 0.014 |
| **Adjunctive Therapy, No. (%)** | | | | | | | |
| ECMO | 7 (1.9%) | 0 (0%) | 3 (1.5%) | | 4 (5.6%) | | 0.024 |
| Lung recruitment maneuver | 48 (13.0%) | 5 (4.9%) | 24 (12.2%) | | 19 (26.8%) | | <0.0001 |
| Prone | 23 (6.2%) | 2 (1.9%) | 10 (5.1%) | | 11 (15.5%) | | 0.001 |
| Neuromuscular blockade | 159 (43.0%) | 21 (20.4%) | 92 (46.9%) | | 46 (64.8%) | | <0.0001 |

Abbreviations: ARDS, acute respiratory distress syndrome; RR, respiratory rate; PEEP, positive end-expiratory pressure; FiO_2_, inspired fraction of oxygen; PaO_2_, partial pressure of oxygen; PaO_2_/FIO_2_, partial pressure of oxygen to fraction of inspired oxygen; P_plat_, plateau pressure; VT, tidal volume; PIP, peak inspiratory pressure; ECMO, extracorporeal membrane oxygenation; sd , standard deviation; IQR (interquartile range); ABG, Artery blood gas .

^a^ ARDS severity determined from worst partial pressure of oxygen to fraction of inspired oxygen ratio within first 24 hours following ARDS diagnosis.

^b^ *P* value represents comparisons across the ARDS severity categories for each variable.
