# Supplementary material for: A combination of oxygenation and driving pressure can provide valuable information in predicting the risk of mortality in ARDS patients
Source: PLoS One. 2023 Dec 13;18(12):e0295261. doi: 10.1371/journal.pone.0295261 (PMC10718417; doi:10.1371/journal.pone.0295261)
Supplement: S3 Table — (DOCX) [file pone.0295261.s003.docx]

**S3 Table. Characteristics of ARDS patients between DP≦14 and DP＞14**

| **Characteristics** | **DP≦14** | **DP＞14** | ***P* Value^a^** |
| --- | --- | --- | --- |
|  | N=211 /318(66.4%) | N=107/318 (33.6%) |  |
| Age (years) | 68.6 ± 15.8 | 67.2 ± 18.8 | 0.486 |
| Male, No. (%) | 141 (66.8%) | 74 (69.2%) | 0.674 |
| BMI (kg/m^2^) | 23.7 ± 4.9 | 24.4 ± 5.2 | 0.220 |
| **Admission source, No. (%)** |  |  |  |
| Emergency Room (ER) | 172 (81.5%) | 92 (86.0%) | 0.604 |
| Non-Emergency Room | 39 (18.5%) | 15 (14.0%) |  |
| **Type of ICU, No. (%)** |  |  |  |
| Medical | 178 (84.4%) | 77 (72.0%) | 0.009 |
| Surgical | 33 (15.6%) | 30 (28.0%) |  |
| **Severity scores** |  |  |  |
| APACHE ll score | 27.7 ± 6.8 | 28.7 ± 6.8 | 0.192 |
| SOFA score, Day 1 | 10.0 ± 3.4 | 10.9 ± 3.7 | 0.030 |
| SOFA score, Day 3 | 8.8 ± 3.9 | 9.8 ± 4.7 | 0.056 |
| SOFA score, Day 7 | 7.3 ± 4.0 | 8.0 ± 4.7 | 0.254 |
| **ARDS Hypoxia severity** |  |  |  |
| Mild | 68 (32.2%) | 27 (25.2%) | 0.432 |
| Moderate | 105 (49.8%) | 58 (54.2%) |  |
| Severe | 38 (18.0%) | 22 (20.6%) |  |
| **Comorbidities, No. (%)** |  |  |  |
| Cardiovascular disease | 59 (28.0%) | 31 (29.0%) | 0.850 |
| Cerebrovascular disease | 47 (22.3%) | 21 (19.6%) | 0.586 |
| Dementia | 17 (8.1%) | 9 (8.4%) | 0.913 |
| Chronic pulmonary disease | 42 (20.0%) | 19 (17.8%) | 0.646 |
| Rheumatic disease | 17 (8.1%) | 7 (6.5%) | 0.629 |
| Hepatic disease | 36 (17.1%) | 18 (16.8%) | 0.957 |
| Diabetes mellitus | 88 (41.7%) | 40 (37.4%) | 0.458 |
| Renal disease | 71 (33.6%) | 39 (36.4%) | 0.620 |
| Malignancy | 72 (34.1%) | 48 (44.9%) | 0.062 |
| Charlson Comorbidity Index (CCI) | 3.6 ± 2.8 | 3.9 ± 3.2 | 0.378 |
| **Etiology of ARDS, No. (%)** |  |  |  |
| **Pulmonary, No. (%)** |  |  |  |
| Pneumonia | 145 (68.7%) | 76 (71.0%) | 0.673 |
| Aspiration | 23 (10.9%) | 8 (7.5%) | 0.331 |
| Pulmonary contusion | 3 (1.4%) | 2 (1.9%) | 0.762 |
| **Extrapulmonary, No. (%)** |  |  |  |
| Sepsis (non-pulmonary source) | 24(11.4%) | 16(15.0%) | 0.363 |
| Trauma or hemorrhagic shock | 2(0.9%) | 1(0.9%) | 0.991 |
| Pancreatitis | 2(0.9%) | 2(1.9%) | 0.486 |
| TRALI | 8(3.8%) | 1(0.9%) | 0.147 |
| **Clinical outcomes** |  |  |  |
| ICU length of stay | 13 (8-21) | 15 (8-24) | 0.630 |
| Hospital length of stay | 29 (18-44) | 33 (18-51) | 0.153 |
| Ventilator-day | 13 (7-21) | 15 (7-28) | 0.170 |
| **Discharge Status** |  |  |  |
| Survival | 140 (66.4%) | 51 (47.7%) | 0.001 |
| Mortality | 71 (33.6%) | 56 (52.3%) |  |

Abbreviations: ARDS, acute respiratory distress syndrome; BMI, Body Mass Index*;* ICU, intensive care unit; APCHE II, Acute Physiology And Chronic Health Evaluation II; SOFA, The sequential organ failure assessment score; CCI, Charlson comorbidity index; TRALI, transfusion-related acute lung injury; sd , standard deviation; IQR (interquartile range) ;DP, driving pressure.

^a^ *P* value represents comparisons between the DP≦14 and DP＞14 ARDS patients.
