# Supplementary material for: A combination of oxygenation and driving pressure can provide valuable information in predicting the risk of mortality in ARDS patients
Source: PLoS One. 2023 Dec 13;18(12):e0295261. doi: 10.1371/journal.pone.0295261 (PMC10718417; doi:10.1371/journal.pone.0295261)
Supplement: S4 Table — (DOCX) [file pone.0295261.s004.docx]

**S4 Table**.**Mechanical ventilation and adjunctive therapy between DP≦14 and**

**DP＞14**

| **Characteristics** | **DP≦14** | **DP＞14** | ***P* Value^a^** |
| --- | --- | --- | --- |
|  | **N=211 (66.4%)** | **N=107 (33.6%)** |  |
| **Ventilator settings**, **first day of ARDS(Day0)** |  |  |  |
| **Mode, No. (%)** |  |  |  |
| Volume -targeted | 190 (90.0%) | 88 (82.2%) | 0.047 |
| Pressure-targeted | 21 (10.0%) | 19 (17.8%) |  |
| FiO_2_ (%) | 0.6 ± 0.2 | 0.7 ± 0.2 | 0.021 |
| PaO_2_ (mmHg) | 104.3 ± 57.7 | 100.1 ± 40.9 | 0.501 |
| P/F ratio (%) | 178.9 ± 93.6 | 158.4 ± 71.1 | 0.047 |
| PEEP (cmH_2_o) | 9.0 ± 3.2 | 8.9 ± 5.8 | 0.799 |
| Tidal Volume (vt/cc/kg) | 7.3 ± 1.4 | 7.3 ± 1.5 | 0.997 |
| RR (1breath/min ) | 22.2 ± 5.0 | 22.5 ± 6.0 | 0.627 |
| PIP (mmHg) | 23.4 ± 4.3 | 27.7 ± 5.2 | <0.0001 |
| P_plat_ (cmH_2_o) | 19.9 ± 3.9 | 25.2 ± 4.9 | <0.0001 |
| Driving pressure (cmH_2_o) | 11.0 ± 2.4 | 16.8 ± 4.1 | <0.0001 |
| Compliance (cmH_2_o) | 39.7 ± 12.5 | 26.7 ± 9.0 | <0.0001 |
| **Artery blood gas(ABG)-patient in ICU 24hrs** |  |  |  |
| pH | 7.4 ± 0.1 | 7.3 ± 0.1 | 0.012 |
| PaO_2_ (mmHg) | 124.7 ± 117.2 | 115.5 ± 61.3 | 0.444 |
| PaCO_2_ (mmHg) | 38.9 ± 10.2 | 45.4 ± 43.2 | 0.124 |
| HCO_3_ | 22.0 ± 5.4 | 22.0 ± 6.3 | 0.918 |
| **Adjunctive Therapy, No. (%)** |  |  |  |
| ECMO | 1 (0.5%) | 5 (4.7%) | 0.009 |
| Lung recruitment maneuver | 31 (14.7%) | 13 (12.1%) | 0.535 |
| Prone position ventilation | 12 (5.7%) | 7 (6.5%) | 0.761 |
| Neuromuscular blockade | 84 (39.8%) | 49 (45.8%) | 0.307 |

Abbreviations: ARDS, acute respiratory distress syndrome; RR, respiratory rate; PEEP, positive end-expiratory pressure; FiO2, inspired fraction of oxygen; PaO2, partial pressure of oxygen; PaO2/FIO2, partial pressure of oxygen to fraction of inspired oxygen; Pplat, plateau pressure; VT, tidal volume; PIP, peak inspiratory pressure; ECMO, extracorporeal membrane oxygenation; sd , standard deviation; IQR (interquartile range); ABG, Artery blood gas.

^a^ P value represents comparisons between the DP≦14 and DP＞14 ARDS patients.
