# Supplementary material for: A combination of oxygenation and driving pressure can provide valuable information in predicting the risk of mortality in ARDS patients
Source: PLoS One. 2023 Dec 13;18(12):e0295261. doi: 10.1371/journal.pone.0295261 (PMC10718417; doi:10.1371/journal.pone.0295261)
Supplement: Attachment — Submitted filename: PONE-D-23-11371.pdf [file pone.0295261.s006.pdf]

PLOS ONE

A combination of oxygenation and driving pressure can provide valuable information in predicting the risk of mortality in ARDS patients.

--Manuscript Draft--

**Manuscript Number:**	PONE-D-23-11371
**Article Type:**	Research Article
**Full Title:**	A combination of oxygenation and driving pressure can provide valuable information in predicting the risk of mortality in ARDS patients.
**Short Title:**	Combing oxygenation driving pressure better predict risk of death of ARDS patients
**Corresponding Author:**	Ming-Cheng Chan, M.D.<br>National Yang-Ming University<br>Taipei, TAIWAN
**Keywords:**	acute respiratory distress syndrome, lung protective strategy, driving pressure, epidemiology.
**Abstract:**	Background: Acute respiratory distress syndrome (ARDS) is a common life-threatening condition in critically ill patients. It is also an important public health issue because it can cause substantial mortality and health care burden worldwide. The objective of this study was to investigate the incidence and outcomes of ARDS patients in a medical center in Taiwan.<br>Methods: This was a single center, observational study which analyzed prospectively collected data from adults in 6 ICUs at Taichung Veterans General Hospital in Taiwan from October 1st2018 to September 30th 2019. Patients needing invasive mechanical ventilation and meeting the Berlin definition criteria were included for analysis.<br>Results: A total of 1,788 subjects were screened in 6 adult ICUs and 370 patients fulfilled the criteria of ARDS in the first 24 hours of ICU admission. Among these patients, the incidence of ARDS was 20.8% and the overall hospital mortality rate was 42.2%. The mortality rates of mild, moderate and severe ARDS were 35.9%, 43.9% and 46.5%, respectively. In a multivariate logistic regression model, an increase of 1 cmH2O driving pressure was associated with an 11% increase in the mortality rate (adjusted odds ratio 1.100, 95% confidence interval 1.027- 1.179, p=0.007). Both oxygenation and driving pressure were independently associated with mortality in ARDS. Patients with worse oxygenation and a higher driving pressure had the highest hospital mortality rate (p<0.0001).<br>Conclusions: ARDS is common in the ICU and the mortality rate remains high. Combining oxygenation and respiratory mechanics may better predict the outcomes of these ARDS patients.
**Order of Authors:**	Yu-Yi Yu, RN
	Wei-Fan Ou, M.D.
	Jia-Jun Wu, M.D.
	Han-Shui Hsu, M.D., PhD
	Chieh-Liang Wu, M.D., PhD
	Kuang-Yao Yang, M.D., PhD
	Ming-Cheng Chan, M.D.,PhD
**Additional Information:**	
**Question**	**Response**
**Financial Disclosure**<br><br>Enter a financial disclosure statement that describes the sources of funding for the work included in this submission. Review	This study was supported by Taichung Veterans General Hospital (TCVGH-1104101C). The funders had no role in study design, data collection and analysis, decision to publish, or preparation of the manuscript.

Powered by Editorial Manager® and ProduXion Manager® from Aries Systems Corporation

the submission guidelines for detailed requirements. View published research articles from *PLOS ONE* for specific examples.<br><br>This statement is required for submission and **will appear in the published article** if the submission is accepted. Please make sure it is accurate.<br><br>**Unfunded studies**<br>Enter: *The author(s) received no specific funding for this work.*<br><br>**Funded studies**<br>Enter a statement with the following details:<br>• Initials of the authors who received each award<br>• Grant numbers awarded to each author<br>• The full name of each funder<br>• URL of each funder website<br>• Did the sponsors or funders play any role in the study design, data collection and analysis, decision to publish, or preparation of the manuscript?<br>• **NO** - Include this sentence at the end of your statement: *The funders had no role in study design, data collection and analysis, decision to publish, or preparation of the manuscript.*<br>• **YES** - Specify the role(s) played.<br><br>* typeset	
**Competing Interests**<br><br>Use the instructions below to enter a competing interest statement for this submission. On behalf of all authors, disclose any competing interests that could be perceived to bias this work—acknowledging all financial support and any other relevant financial or non-financial competing interests.<br><br>This statement is required for submission and **will appear in the published article** if the submission is accepted. Please make sure it is accurate and that any funding sources listed in your Funding Information later in the submission form are also	The authors have declared that no competing interests exist.

declared in your Financial Disclosure statement.<br><br>View published research articles from *PLOS ONE* for specific examples.<br><br>**NO authors have competing interests**<br><br>Enter: *The authors have declared that no competing interests exist.*<br><br>**Authors with competing interests**<br><br>Enter competing interest details beginning with this statement:<br><br>*I have read the journal's policy and the authors of this manuscript have the following competing interests: [insert competing interests here]*<br><br>* typeset	
**Ethics Statement**<br><br>Enter an ethics statement for this submission. This statement is required if the study involved:<br><br>• Human participants<br>• Human specimens or tissue<br>• Vertebrate animals or cephalopods<br>• Vertebrate embryos or tissues<br>• Field research<br><br>Write "N/A" if the submission does not require an ethics statement.<br><br>General guidance is provided below. Consult the submission guidelines for detailed instructions. **Make sure that all information entered here is included in the Methods section of the manuscript.**	This study was approved by the Institutional Review Board of Taichung Veterans General Hospital Taiwan (IRB number: CE20049B). Given that the research was limited to the secondary use of data previously collected during daily practice and the patients' identity were completely unidentifiable, the informed consent was hence waived

**Format for specific study types**<br><br>**Human Subject Research (involving human participants and/or tissue)**<br>• Give the name of the institutional review board or ethics committee that approved the study<br>• Include the approval number and/or a statement indicating approval of this research<br>• Indicate the form of consent obtained (written/oral) or the reason that consent was not obtained (e.g. the data were analyzed anonymously)<br><br>**Animal Research (involving vertebrate animals, embryos or tissues)**<br>• Provide the name of the Institutional Animal Care and Use Committee (IACUC) or other relevant ethics board that reviewed the study protocol, and indicate whether they approved this research or granted a formal waiver of ethical approval<br>• Include an approval number if one was obtained<br>• If the study involved *non-human primates*, add *additional details* about animal welfare and steps taken to ameliorate suffering<br>• If anesthesia, euthanasia, or any kind of animal sacrifice is part of the study, include briefly which substances and/or methods were applied<br><br>**Field Research**<br><br>Include the following details if this study involves the collection of plant, animal, or other materials from a natural setting:<br>• Field permit number<br>• Name of the institution or relevant body that granted permission	
**Data Availability**<br><br>Authors are required to make all data underlying the findings described fully available, without restriction, and from the time of publication. PLOS allows rare exceptions to address legal and ethical concerns. See the PLOS Data Policy and FAQ for detailed information.	Yes - all data are fully available without restriction

A Data Availability Statement describing where the data can be found is required at submission. Your answers to this question constitute the Data Availability Statement and **will be published in the article**, if accepted.<br><br>**Important:** Stating 'data available on request from the author' is not sufficient. If your data are only available upon request, select 'No' for the first question and explain your exceptional situation in the text box.<br><br>Do the authors confirm that all data underlying the findings described in their manuscript are fully available without restriction?	
**Describe where the data may be found in full sentences. If you are copying our sample text, replace any instances of XXX with the appropriate details.**<br><br>• If the data are **held or will be held in a public repository**, include URLs, accession numbers or DOIs. If this information will only be available after acceptance, indicate this by ticking the box below. For example: *All XXX files are available from the XXX database (accession number(s) XXX, XXX.*).<br>• If the data are all contained **within the manuscript and/or Supporting Information files**, enter the following: *All relevant data are within the manuscript and its Supporting Information files.*<br>• If neither of these applies but you are able to provide **details of access elsewhere**, with or without limitations, please do so. For example:<br><br>*Data cannot be shared publicly because of [XXX]. Data are available from the XXX Institutional Data Access / Ethics Committee (contact via XXX) for researchers who meet the criteria for access to confidential data.*<br><br>*The data underlying the results presented in the study are available from (include the name of the third party*	NA

and contact information or URL).<br>• This text is appropriate if the data are owned by a third party and authors do not have permission to share the data.<br><br>* typeset	
Additional data availability information:	Tick here if the URLs/accession numbers/DOIs will be available only after acceptance of the manuscript for publication so that we can ensure their inclusion before publication.

Powered by Editorial Manager® and ProduXion Manager® from Aries Systems Corporation

Manuscript

Click here to access/download;Manuscript;the risk of mortality in ARDS patients..docx

A combination of oxygenation and driving pressure can provide valuable information in predicting the risk of mortality in ARDS patients.

Yu-Yi Yu[1,2,], Wei-Fan Ou[3], Jia-Jun Wu[3,4], Han-Shui Hsu[1,5], Chieh-Laing Wu[2,8],

Kuang-Yao Yang[1,6,7], Ming-Cheng Chan[2,3,8*]

[1]Institute of Emergency and Critical Care Medicine, National Yang Ming Chiao Tung University, Taipei, Taiwan.

[2]Department of Critical Care Medicine, Taichung Veterans General Hospital, Taichung, Taiwan.

[3]Division of Chest Medicine, Department of Internal Medicine, Taichung Veterans General Hospital, Taichung, Taiwan.

[4] Division of Pulmonary Medicine, Department of Internal Medicine, Chung Shan Medical University Hospital, Taichung, Taiwan.

[5]Department of Thoracic Surgery, Taipei Veterans General Hospital, Taipei, Taiwan.

[6]Department of Chest Medicine, Taipei Veterans General Hospital, Taipei, Taiwan.

[7]School of Medicine, National Yang Ming Chiao Tung University, Taipei, Taiwan.

[8]School of Post Baccalaureate Medicine, National Chung Hsing University, Taichung, Taiwan.

Authors:

Yu-Yi Yu, RN, Email: propanolo0829@gmail.com

Address: 1650 Taiwan Boulevard Sect. 4, Taichung, Taiwan 40705

[1]Institute of Emergency and Critical Care Medicine, National Yang Ming Chiao Tung

University, Taipei, Taiwan.

[2]Department of Critical Care Medicine, Taichung Veterans General Hospital, Taichung, Taiwan.

Wei-Fan Ou, MD, Email: spmanner@hotmail.com

Address: 1650 Taiwan Boulevard Sect. 4, Taichung, Taiwan 40705

[3]Division of Chest Medicine, Department of Internal Medicine, Taichung Veterans General Hospital, Taichung, Taiwan.

Jia-Jun Wu , MD, Email: Jameswu19861114@hotmail.com

Address: 1650 Taiwan Boulevard Sect. 4, Taichung, Taiwan 40705

[3]Division of Chest Medicine, Department of Internal Medicine, Taichung Veterans General Hospital, Taichung, Taiwan.

[4]Division of Pulmonary Medicine, Department of Internal Medicine, Chung Shan Medical University Hospital, Taichung, Taiwan

Han-Shui Hsu, MD, PhD, Email: hsuhs@vghtpe.gov.tw

Address: No. 155, Sec. 2, Linong Street, Taipei, 11221, Taiwan

[1]Institute of Emergency and Critical Care Medicine, National Yang Ming Chiao Tung University, Taipei, Taiwan.

[5]Department of Thoracic Surgery, Taipei Veterans General Hospital, Taipei, Taiwan.

Chieh-Liang Wu, MD, PhD, Email: cljeff.wu@gmail.com

Address: 1650 Taiwan Boulevard Sect. 4, Taichung, Taiwan 40705

[2]Department of Critical Care Medicine, Taichung Veterans General Hospital, Taichung, Taiwan.

[8]School of Post Baccalaureate Medicine, National Chung Hsing University, Taichung, Taiwan.

Kuang-Yao Yang, MD, PhD, Email: kyyang@vghtpe.gov.tw

Address: No. 155, Sec. 2, Linong Street, Taipei, 11221, Taiwan

[1]Institute of Emergency and Critical Care Medicine, National Yang Ming Chiao Tung University, Taipei, Taiwan.

[6]Department of Chest Medicine, Taipei Veterans General Hospital, Taipei, Taiwan.

[7]School of Medicine, National Yang Ming Chiao Tung University, Taipei, Taiwan.

Ming-Cheng Chan, MD, PhD, Email: mingcheng.chan@gmail.com

Address: 1650 Taiwan Boulevard Sect. 4, Taichung, Taiwan 40705

[2]Department of Critical Care Medicine, Taichung Veterans General Hospital, Taichung, Taiwan.

[3]Division of Chest Medicine, Department of Internal Medicine, Taichung Veterans General Hospital, Taichung, Taiwan.

[8]School of Post Baccalaureate Medicine, National Chung Hsing University, Taichung, Taiwan.

*Corresponding Author:

Ming-Cheng Chan MD PhD

Department of Critical Care Medicine, Taichung Veterans General Hospital, 1650 Taiwan Boulevard Sect. 4, Taichung, Taiwan, 40705

Tel +884-4-23592525 ext. 3910

Fax +886-4-23596975

E-mail: mingcheng.chan@gmail.com

Conflicts of interest

On behalf of all authors, the corresponding author states that there are no conflicts of interest.

Abstract

Background: Acute respiratory distress syndrome (ARDS) is a common life-threatening condition in critically ill patients. It is also an important public health issue because it can cause substantial mortality and health care burden worldwide. The objective of this study was to investigate the incidence and outcomes of ARDS patients in a medical center in Taiwan.

Methods: This was a single center, observational study which analyzed prospectively collected data from adults in 6 ICUs at Taichung Veterans General Hospital in Taiwan from October 1st2018 to September 30th 2019. Patients needing invasive mechanical ventilation and meeting the Berlin definition criteria were included for analysis.

Results: A total of 1,788 subjects were screened in 6 adult ICUs and 370 patients fulfilled the criteria of ARDS in the first 24 hours of ICU admission. Among these patients, the incidence of ARDS was 20.8% and the overall hospital mortality rate was 42.2%. The mortality rates of mild, moderate and severe ARDS were 35.9%, 43.9% and 46.5%, respectively. In a multivariate logistic regression model, an increase of 1 cmH_2O driving pressure was associated with an 11% increase in the mortality rate (adjusted odds ratio 1.100, 95% confidence interval 1.027- 1.179, p=0.007). Both oxygenation and driving pressure were independently associated with mortality in ARDS. Patients with worse oxygenation and a higher driving pressure had the highest

hospital mortality rate ($p<0.0001$).

Conclusions: ARDS is common in the ICU and the mortality rate remains high. Combining oxygenation and respiratory mechanics may better predict the outcomes of these ARDS patients.

Key words: acute respiratory distress syndrome, lung protective strategy, driving pressure, epidemiology.

Introduction

Acute respiratory distress syndrome (ARDS) is a life-threatening condition which may result from a variety of pulmonary (e.g., pneumonia and aspiration) and extra-pulmonary causes (e.g., non-pulmonary sepsis, trauma and pancreatitis). ARDS is characterized by acute, diffuse alveolar inflammation and flooding, leading to increased capillary permeability, which results in lung tissue edema and loss of aeration. Clinically, patients with ARDS often present with hypoxemia, pulmonary congestion, and decreased respiratory compliance. Patients with ARDS often need invasive mechanical ventilation to rescue their hypoxemia. Although there have been recent advances in the management of ARDS, including protective mechanical ventilation [1], prone positioning [2] and extracorporeal life support [3], the mortality rate remains high [4]. In addition, patients who survive ARDS may also suffer from significant sustained disabilities, both physically and psychologically, leading to a decreased quality of life and even increased mortality in later years [5, 6].

ARDS is common in critically ill patients who are admitted to intensive care units (ICUs), but often goes unrecognized and undertreated [4]. In the United States, ARDS affects approximately 200,000 patients per year and results in about 75,000 deaths annually [7]. ARDS is also an important public health issue as it is a common cause of death for severe respiratory system infections, especially influenza [8] and

coronavirus disease 2019 (COVID-19) [9, 10].

In Taiwan, the epidemiological data, patterns of care and outcomes of patients with ARDS are not clear. Given that ARDS has as an important impact on public health burden, we conducted a prospective observational study to investigate its incidence and factors associated with the outcomes of critically ill patients with ARDS.

Method

Patient and study design

This was an observational study of six adult ICUs in a single tertiary referral institute in central Taiwan. The ICUs were functionally divided into medical, respiratory, neurological, surgical, cardiovascular and trauma. We retrospectively analyzed the data of critically ill patients with ARDS who were admitted to the adult ICUs of Taichung Veterans General Hospital (TCVGH) from the 1st October 2018 to the 30th September 2019. Adult patients (age >20 years old) who were admitted to the ICUs needing invasive mechanical ventilation and who met the Berlin definition criteria for ARDS [11] were included for analysis. Patients with ARDS were identified by a respiratory therapist and verified by two critical care physicians with a respiratory specialty. During the study period, we prospectively collected data for a quality

improvement program via a clinical audit of daily practice in patients with acute respiratory failure who needed mechanical ventilation in the ICUs. This study was approved by the Institutional Review Board of Taichung Veterans General Hospital Taiwan (IRB number: CE20049B). Data were collected and accessed after IRB approval on February 21, 2020. Given that the research was limited to the secondary use of data previously collected during daily practice and the fact that the patients' identities were completely anonymized, the requirement for informed consent from the study subjects was waived by the IRB of Taichung Veterans General Hospital Taiwan due to no more than minimal risk to the subjects. All methods were carried out in accordance with the IRB's guidelines and regulations.

Data collection

All data was collected from medical records, including the patients' demographic data, comorbidities, and basic laboratory tests. Disease severity scores, including Acute Physiology and Chronic Health Evaluation II (APACHE II) [12], and Sequential Organ Failure Assessment (SOFA) scores [13] were determined on the first, third and seventh days of ICU admission. Mechanical ventilation parameters were also collected, including fraction of inspired oxygen (FiO_2), positive end-expiratory pressure (PEEP, cmH_2O), tidal volume (VT) adjusted by predicted body weight

(PBW), plateau pressure (P_{plat}) and peak airway pressure (P_{peak}) on the first three days of ICU admission. P_{plat} was measured at end-inspiration by a 0.5 to 1 second inspiration hold on a mechanical ventilator within 24 hours of the initiation of mechanical ventilation. Driving pressure was calculated as the difference between P_{plat} and PEEP [14].

Information on major comorbidities, including cardiovascular disease, cerebrovascular disease, dementia, chronic pulmonary disease, rheumatic disease and malignancy was collected. The Charlson comorbidity index (CCI) [15] was calculated using the International Classification of Disease 10th Revision [16] diagnosis codes from the patients' medical records. Diabetes was defined by medical history and a laboratory exam with HbA1c >6.5%. The severity of ARDS was classified by thePaO_2/FiO_2 (P/F)ratio according to the Berlin definition [11]. Mechanical ventilator parameters, including mode, FiO_2, VT, PEEP, respiratory rate (RR), peak inspiratory pressure (PIP), and P_{plat} were collected in the first 24 hours of ICU admission. ARDS severity was determined using the worst partial pressure of oxygen to fraction of inspired oxygen ratio within the first 24 hours following ARDS diagnosis.

Statistical analysis

Data is presented as frequency (percentages) for categorical variables and as mean ±

standard deviation or median (interquartile range [IQR]) for continuous variables. Differences between alive and dead groups were determined using the Student's t-test and the Mann-Whitney U test for continuous variables and the Chi-squared test for categorical variables. One-way analysis of variance was used to compare variables among the three ARDS severity groups. Kaplan-Meier analysis was performed to test the mortality correlation of driving pressure above or below 14 cmH_2O in different ARDS severities. The log-rank test was used to compare mortality between two groups. A multivariate logistic regression model was conducted to identify independent variables that predicted mortality. Statistical significance was set at a two-sided p value of <0.05. All data were analyzed using SPSS software version 22.0 (SPSS Inc., IBM Corp, Armonk, NY, USA).

Results

Patient enrollment and clinical features

Of 1,778 patients with acute respiratory failure who needed invasive mechanical ventilation, 370 (20.8%) fulfilled the criteria of ARDS in the first 24 hours of ICU admission (Figure 1). The hospital mortality of patients with ARDS was 42.2%, while in those without ARDS it was just 21.0%. In this cohort of ARDS patients, the average age was 67.7 years old. There were more male (68.1%) than female (31.9%)

patients. Most of these ARDS patients were admitted via the emergency room (81.9%) and there were more in the medical (79.7%) ICU compared with the surgical (20.3%) ICU. The overall disease severity in this cohort of ARDS patients was high in terms of APACHE II and SOFA scores at admission and on the following ICU days. Comorbidities were common among ARDS patients. Diabetes, renal disease and malignancy were the most common comorbidities in this cohort. The etiology of ARDS came primarily from pulmonary (80.5%) causes as opposed to extrapulmonary (19.5%) causes. The leading causes of ARDS were pneumonia and aspiration for pulmonary ARDS and sepsis for extrapulmonary ARDS. ADRS patients also consumed a substantial amount of medical resources, with long ICU stays (13, 8-22 days), hospital stays (29.5, 18-46 days) and ventilator days (13, 7-24 days) (Table 1).

ARDS severity

In this cohort of 370 patients, 103 (27.8%) had mild ARDS, 196 (53%) had moderate and 71 had severe (19.2%). The increasing severity of ARDS was paralleled by a worsening of the APCHE II and SOFA scores at admission and on the following ICU days. There was no significant difference in age, sex, admission source, ICU type, CCI and etiology among the three severity groups. Patients with higher severity ARDS had a higher body mass index (BMI). Among the co-morbidities, dementia was

more common in milder ARDS patients. The clinical outcomes, including ICU stay, hospital stay, ventilator-days and hospital mortality, were not significantly different among the three groups (Table S1).

Mechanical ventilation and adjunctive treatments in ARDS

Mechanical ventilation management on the first day of ICU admission varies with ARDS severity. Volume-targeted ventilation (82.4%) was preferred in this cohort of patients. For the ventilator settings, FiO_2, PEEP and respiratory rate increased with ARDS severity, whereas VT decreased. The measured pressures, including PIP, P_{plat} and driving pressure changed in parallel with ARDS severity. The average VT was 7.5 ± 1.6 mL/kg PBW and 65.5% of these patients had ≤8mL/kg PBW. P_{plat} was measured in 84.3% of patients and the average P_{plat} was 21.5 ± 5.2 cm H_2O; 96.2% of these patients had ≤30 cm H_2O. A total of 64.4% of these patients received protective mechanical ventilation as defined by a VT of ≤8 mL/kg PBW and a P_{plat} ≤30 cm H_2O. The average driving pressure was 12.9 ± 4.1 cm H_2O and 66.4% of these patients had ≤14 cm H_2O. Positive end-expiratory pressure was relatively low with the average PEEP 8.8 ± 4.1 cm H_2O in all patients and 10.2 ± 3.0 cm H_2O in severe patients.

The use of adjunctive treatments in patients with ARDS was relatively low but increased with ARDS severity. In patients with severe ARDS, the percentages of

patients using extracorporeal membrane oxygenation, prone position, and recruitment maneuver were 5.6% , 15.5%, and 26.8%, respectively. Neuromuscular blockade was used in 43.0% of all patients and in 64.8% of severe ARDS patients (Table S2).

Characteristics of ARDS patients according to driving pressure

We further analyzed the characteristics of patients by driving pressure. Driving pressure at admission was available in 318 (85.9%) of the patients. Those with a driving pressure >14 cmH_2O had a significantly lower survival rate (33.6% vs 52.3%, $p=0.001$) (Table S3). There was no significant difference in age, gender, BMI, comorbidities, admission source, type of ICU, etiology, length of ICU stay, length of hospital stay or ventilator days between groups of driving pressure (DP) $\leq$14cmH_2O and DP >14 cmH_2O. In ARDS patients with different DPs, the ventilator parameters including PIP and P_{plat}, were significantly lower in the DP $\leq$14cmH_2O group compared with the DP >14 cmH_2O group ($p<0.0001$) (Table S4).

Mortality of ARDS

The hospital mortality in this ARDS cohort was 42.2% and the hospital mortality rate was paralleled by ARDS severity, which was 35.9% in mild, 43.9% in moderate and 46.5% in severe ARDS. There was no significant difference in age, sex, BMI,

admission source, and type of ICU between the survivors and non-survivors. Non-survivors had higher APACHE II and SOFA scores on the days following ICU admission. Non-survivors also had higher CCI scores than survivors. Among the comorbidities, the incidence of rheumatic disease, hepatic disease and malignancy were higher in the non-survivors, but diabetes was more common in survivors (Table 1). Among mechanical ventilator parameters, VT and PEEP were not significantly different between the survivors and non-survivors. However, PIP, P_{plat} and driving pressure were higher in non-survivors compared with survivors (Table 2). A multivariate regression model was adopted by adjusting for possible confounders, including prone position ventilation, aspiration of gastric content, CCI, mean arterial pressure, BMI, APACHE II score and driving pressure, to evaluate independent risk factors for hospital mortality. Three independent factors were identified, including APACHE II score (adjusted odds ratio (OR) 1.094, 95% confidence interval (CI) 1.045-1.145; $p<0.0001$), CCI (OR 1.12, 95% CI 1.012-1.222, $p=0.27$), and driving pressure (OR 1.100, 95% CI 1.027-1.179, $p=0.007$) (Table 3).

As oxygenation (P/F ratio) and respiratory mechanics (driving pressure) were both associated with mortality in ARDS, we constructed an analysis which combined these two factors. Patients with a driving pressure >14 cm H_2O (Fig. 2A, $p=0.025$) and a P/F ratio ≤150 (Fig.2B, $p=0.002$) had a higher hospital mortality rate. By

combining these factors, patients with worse oxygenation and higher driving pressure had the highest hospital mortality rate (Fig. 2C, p<0.0001).

Discussion

The present study was carried out in 6 ICUs of a referral medical center in central Taiwan. ARDS appears to represent an important health problem, which is both common and has a high mortality, in critically ill patients receiving invasive mechanical ventilation. We found that CCI, driving pressure and APACHE II scores were independent risk factors of mortality, and that a combination of oxygenation status and respiratory mechanics gives better outcome prediction in ARDS patients. Adhesion to contemporary ventilation strategies and adjuncts in ARDS patients remains unsatisfactory. These findings also indicate the potential for improvement in the management of patients with ARDS.

The burden of critical illness is increasing worldwide because of aging populations, natural disasters, conflicts, and higher-risk medical therapies [17]. The number of critically ill patients admitting to ICUs is increasing and most of these patients need mechanical ventilation [18]. In the LUNG SAFE study, approximately a quarter of patients admitted to the ICU and who received mechanical ventilation developed ARDS [4], and there is a geographical variation in the incidence of ARDS, which is probably due to the health care system, medical resource availability, and ethnics. Previous epidemiological studies of ARDS in Taiwan are limited due to the small-size of case series [19] and the imprecise case definition used [20]. As there has

been a substantial evolution in the health care system over the past two decades, there has been the development of a new ARDS definition [11], and epidemiological data on the incidence, pattern of care, and outcome of ARDS patients in Taiwan is urgently needed. Our study demonstrates that the incidence of ARDS remains high, and that the mortality rate of these patients is doubled compared with patients without ARDS. This highlights the fact that critical care teams should pay more attention to the prevention and management of patients with, or at risk of ARDS.

The presence of comorbidities is common in critically ill patients admitted to ICUs. There is a trend that the number of comorbidities increases over time in non-surgical patients needing invasive mechanical ventilation [18]. The presence of chronic illness, such as active neoplasm, hematological neoplasm, and chronic liver failure, are independent risk factors of worse outcomes in ARDS patients [21]. The concept of limiting life-sustaining therapies or measures in critically ill patients varies among Asian countries and regions [22]. Comorbidities are not modifiable and can also serve as a key factor in decisions to forgo life-sustaining treatment [23]. In the present study, we calculated CCI and found that it independently predicted mortality in ARDS patients. Since the majority of decisions to limit care in critically ill patients are made after the development of ARDS, the presence of comorbidities can provide important information when making this decision.

In real-life practice, recognition of ARDS is often delayed or missed, especially in those with mild disease. However, the mortality of ARDS patients remains high even in those with mild ARDS. In this cohort of ARDS patients, the mortality rate of mild disease was 35.9%, which is similar to the LUNG SAFE study, but still much higher than in those without ARDS. The mortality rate is even higher if ARDS worsens over the following days. Pham et al. emphasized the need to pay close attention to those with mild ARDS [24]. Failure to recognize ARDS in a timely fashion can lead to failure to implement strategies that improve survival of ARDS. Timely implementation of lung protection with low VT is fundamental to successful treatment in ARDS [25]. In severe influenza ARDS patients, first VT, shortly after intubation, is associated with increased mortality [26]. In this study, although ARDS was recognized in a timely manner at ICU admission, the outcomes of these patients were not satisfactory. This can be attributed to adherence to lung protection and the application of adjunct therapies. Another important issue for ARDS in real-life practice is that it is often under recognized. The LUNG SAFE study also indicated that a lower ratio of healthcare professionals to ICU patients, was associated with reduced recognition of ARDS. A global epidemiological investigation into the burden of critical illness also suggested that ICU organization has an important effect on the risk of death [27]. Taken together, our results also highlight the importance of timely

identification and proper management of ARDS, both of which may be improved by investing resources to improve the workforce in ICUs.

As hypoxemia is the major clinical presentation of ARDS, the severity of ARDS is traditionally classified by the P/F ratio [28]. As variations in PEEP and FiO_2 levels can impact the P/F ratio, it is now considered that PEEP or continuous positive airway pressure ≥5 cm H_2O is required to define and classify ARDS severity. However, the difference in mortality between mild and severe ARDS was around 10% in LUNG SAFE and our study. Respiratory mechanics, such as P_{plat} [29] and driving pressure [14], are good predictors of outcomes in ARDS patients. In addition, improvement of respiratory mechanics, such as an increase in dynamic driving pressure [30] or a decrease in $PaCO_2$ [31], are associated with improved survival in prone positioning of severe ARDS patients. The respiratory response to prone positioning was more relevant when $PaCO_2$ rather than the P/F ratio was used [32]. In this study, by combining respiratory mechanics and oxygenation status, we found that patients with a driving pressure >14 cm H_2O and a P/F ≤150 have worst outcomes. ARDS is also an inflammatory disease, both locally and systematically, and hyper inflammation is associated with clinical outcomes. We therefore suggest the need to develop a new classification model which combines oxygenation, respiratory mechanics and inflammation parameters and which may better predict outcomes for ARDS patients.

A major strength of this study was that the data was collected prospectively by a quality improvement program for a year. In contrast to previous studies which were conducted over 4 weeks (LUNG SAFE) to 2 months [33], this epidemiological study avoided any seasonal variation in ARDS incidence. This study also had several limitations. First, this was a single center study and is inherently limited for external validation. As the Taiwan National Health Insurance is a single payer system, the hospitals share high homogeneity in their practice patterns and ICU organization. As this was an observational study without intervention, the results of this study are representative of real-life information about the diagnosis and management of ARDS in ICUs. Second, we only included patients with invasive mechanical ventilation within 24 hours of ICU admission. Patients with non-invasive ventilation were not included. These patients either had mild ARDS with a good prognosis or were those designated as ‘do not intubate’ due to their advanced age or end-stage disease. We also did not include ARDS which developed after the first day of ICU mission because most ARDS cases are early onset in ICU admission. However, all these limitations should be taken into account when interpretating the information collected.

Conclusions

ARDS is common among critically ill patients who are admitted to the ICU and who use invasive mechanical ventilation. Hospital mortality remains high and could potentially be improved by adherence to currently available evidence. Both CCI and driving pressure are independent predictors of outcomes in ARDS patients. However, combining oxygenation status and respiratory mechanics may better predict ARDS patients at risk of mortality.

List of abbreviations

Term	Definition
ARDS	*Acute respiratory distress syndrome*
BMI	*Body Mass Index*
ICU	*Intensive care unit*
APCHE II	Acute Physiology and Chronic Health Evaluation II
SOFA	The sequential organ failure assessment score
TRALI	Transfusion-related acute lung injury
CCI	*Charlson Comorbidity Index*
RR	Respiratory rate
PEEP	Positive end-expiratory pressure
FiO_2	Inspired fraction of oxygen
PaO_2	Partial pressure of oxygen
P/F ratio	Ratio of partial pressure of oxygen to fraction of inspired oxygen
P_{plat}	Plateau pressure
VT	Tidal volume
PIP	Peak inspiratory pressure
MAP	Mean Airway Pressure
ABG	Artery blood gas
DP	Driving pressure
NMBAs	Neuromuscular blocking agents
SD	Standard deviation
IQR	Interquartile range
OR	Adjusted odds ratio
CI	Confidence interval

Availability of data and material

The datasets consulted during this study are available from the corresponding author on reasonable request.

References

1. Brower, R.G., M.A. Matthay, A. Morris, D. Schoenfeld, B.T. Thompson, and A. Wheeler. Ventilation with lower tidal volumes as compared with traditional tidal volumes for acute lung injury and the acute respiratory distress syndrome. N Engl J Med, 2000; 342(18): 1301-8.
2. Guerin, C., J. Reignier, J.C. Richard, P. Beuret, A. Gacouin, T. Boulain, et al. Prone positioning in severe acute respiratory distress syndrome. N Engl J Med, 2013; 368(23): 2159-68.
3. Combes, A., D. Hajage, G. Capellier, A. Demoule, S. Lavoue, C. Guervilly, et al. Extracorporeal Membrane Oxygenation for Severe Acute Respiratory Distress Syndrome. N Engl J Med, 2018; 378(21): 1965-1975.
4. Bellani, G., J.G. Laffey, T. Pham, E. Fan, L. Brochard, A. Esteban, et al. Epidemiology, Patterns of Care, and Mortality for Patients With Acute Respiratory Distress Syndrome in Intensive Care Units in 50 Countries. JAMA, 2016; 315(8): 788-800.
5. Herridge, M.S., A.M. Cheung, C.M. Tansey, A. Matte-Martyn, N. Diaz-Granados, F. Al-Saidi, et al. One-year outcomes in survivors of the acute respiratory distress syndrome. N Engl J Med, 2003; 348(8): 683-93.
6. Herridge, M.S., C.M. Tansey, A. Matté, G. Tomlinson, N. Diaz-Granados, A. Cooper, et al. Functional disability 5 years after acute respiratory distress syndrome. N Engl J Med, 2011; 364(14): 1293-304.
7. Rubenfeld, G.D., E. Caldwell, E. Peabody, J. Weaver, D.P. Martin, M. Neff, et al. Incidence and outcomes of acute lung injury. N Engl J Med, 2005; 353(16): 1685-93.
8. Short, K.R., E.J.B.V. Kroeze, R.A.M. Fouchier, and T. Kuiken. Pathogenesis of influenza-induced acute respiratory distress syndrome. Lancet Infect Dis, 2014; 14(1): 57-69.
9. Grasselli, G., T. Tonetti, A. Protti, T. Langer, M. Girardis, G. Bellani, et al. Pathophysiology of COVID-19-associated acute respiratory distress syndrome: a multicentre prospective observational study. Lancet Respir Med, 2020; 8(12): 1201-1208.
10. Ferrando, C., F. Suarez-Sipmann, R. Mellado-Artigas, M. Hernandez, A. Gea, E. Arruti, et al. Clinical features, ventilatory management, and outcome of ARDS caused by COVID-19 are similar to other causes of ARDS. Intensive Care Med, 2020; 46(12): 2200-2211.

11. Force, A.D.T., V.M. Ranieri, G.D. Rubenfeld, B.T. Thompson, N.D. Ferguson, E. Caldwell, et al. Acute respiratory distress syndrome: the Berlin Definition. JAMA, 2012; 307(23): 2526-33.
12. Knaus, W.A., J.E. Zimmerman, D.P. Wagner, E.A. Draper, and D.E. Lawrence. APACHE-acute physiology and chronic health evaluation: a physiologically based classification system. Crit Care Med, 1981; 9(8): 591-7.
13. Vincent, J.L., A. de Mendonça, F. Cantraine, R. Moreno, J. Takala, P.M. Suter, et al. Use of the SOFA score to assess the incidence of organ dysfunction/failure in intensive care units: results of a multicenter, prospective study. Working group on "sepsis-related problems" of the European Society of Intensive Care Medicine. Crit Care Med, 1998; 26(11): 1793-800.
14. Amato, M.B., M.O. Meade, A.S. Slutsky, L. Brochard, E.L. Costa, D.A. Schoenfeld, et al. Driving pressure and survival in the acute respiratory distress syndrome. N Engl J Med, 2015; 372(8): 747-55.
15. Charlson, M.E., P. Pompei, K.L. Ales, and C.R. MacKenzie. A new method of classifying prognostic comorbidity in longitudinal studies: development and validation. J Chronic Dis, 1987; 40(5): 373-83.
16. World Health organixation. International Classification of Diseases. 2022/01/04; Available from: https://www.who.int/standards/classifications/classification-of-diseases.
17. Adhikari, N.K., R.A. Fowler, S. Bhagwanjee, and G.D. Rubenfeld. Critical care and the global burden of critical illness in adults. Lancet, 2010; 376(9749): 1339-46.
18. Mehta, A.B., S.N. Syeda, R.S. Wiener, and A.J. Walkey. Epidemiological trends in invasive mechanical ventilation in the United States: A population-based study. J Crit Care, 2015; 30(6): 1217-21.
19. Jerng, J.S., C.J. Yu, Y.S. Liaw, H.D. Wu, H.C. Wang, P.H. Kuo, et al. Clinical spectrum of acute respiratory distress syndrome in a tertiary referral hospital: etiology, severity, clinical course, and hospital outcome. J Formos Med Assoc, 2000; 99(7): 538-43.
20. Chen, W., Y.Y. Chen, C.F. Tsai, S.C. Chen, M.S. Lin, L.B. Ware, et al. Incidence and Outcomes of Acute Respiratory Distress Syndrome: A Nationwide Registry-Based Study in Taiwan, 1997 to 2011. Medicine (Baltimore), 2015; 94(43): e1849.
21. Laffey, J.G., G. Bellani, T. Pham, E. Fan, F. Madotto, E.K. Bajwa, et al. Potentially modifiable factors contributing to outcome from acute respiratory distress syndrome: the LUNG SAFE study. Intensive Care Med, 2016; 42(12): 1865-1876.

22. Phua, J., G.M. Joynt, M. Nishimura, Y. Deng, S.N. Myatra, Y.H. Chan, et al. Withholding and withdrawal of life-sustaining treatments in low-middle-income versus high-income Asian countries and regions. Intensive Care Med, 2016; 42(7): 1118-27.
23. Lautrette, A., M. Garrouste-Orgeas, P.M. Bertrand, D. Goldgran-Toledano, S. Jamali, V. Laurent, et al. Respective impact of no escalation of treatment, withholding and withdrawal of life-sustaining treatment on ICU patients' prognosis: a multicenter study of the Outcomerea Research Group. Intensive Care Med, 2015; 41(10): 1763-72.
24. Pham, T., A. Serpa Neto, P. Pelosi, J.G. Laffey, C. De Haro, J.A. Lorente, et al. Outcomes of Patients Presenting with Mild Acute Respiratory Distress Syndrome: Insights from the LUNG SAFE Study. Anesthesiology, 2019; 130(2): 263-283.
25. Needham, D.M., T. Yang, V.D. Dinglas, P.A. Mendez-Tellez, C. Shanholtz, J.E. Sevransky, et al. Timing of low tidal volume ventilation and intensive care unit mortality in acute respiratory distress syndrome. A prospective cohort study. Am J Respir Crit Care Med, 2015; 191(2): 177-85.
26. Chan, M.C., W.C. Chao, S.J. Liang, C.H. Tseng, H.C. Wang, Y.C. Chien, et al. First tidal volume greater than 8 mL/kg is associated with increased mortality in complicated influenza infection with acute respiratory distress syndrome. J Formos Med Assoc, 2019; 118: 378-385.
27. Vincent, J.L., J.C. Marshall, S.A. Namendys-Silva, B. François, I. Martin-Loeches, J. Lipman, et al. Assessment of the worldwide burden of critical illness: the intensive care over nations (ICON) audit. Lancet Respir Med, 2014; 2(5): 380-6.
28. Bernard, G.R., A. Artigas, K.L. Brigham, J. Carlet, K. Falke, L. Hudson, et al. The American-European Consensus Conference on ARDS. Definitions, mechanisms, relevant outcomes, and clinical trial coordination. Am J Respir Crit Care Med, 1994; 149(3): 818-24.
29. Chan, M.C., J.S. Tseng, J.T. Chiu, K.H. Hsu, S.J. Shih, C.Y. Yi, et al. Prognostic value of plateau pressure below 30 cm H2O in septic subjects with acute respiratory failure. Respir Care, 2015; 60(1): 12-20.
30. Kao, K.C., K.W. Chang, M.C. Chan, S.J. Liang, Y.C. Chien, H.C. Hu, et al. Predictors of survival in patients with influenza pneumonia-related severe acute respiratory distress syndrome treated with prone positioning. Ann Intensive Care, 2018; 8(1): 94.
31. Gattinoni, L., F. Vagginelli, E. Carlesso, P. Taccone, V. Conte, D. Chiumello, et al. Decrease in PaCO2 with prone position is predictive of improved

outcome in acute respiratory distress syndrome. Crit Care Med, 2003; 31(12): 2727-33.

32. Charron, C., X. Repesse, K. Bouferrache, L. Bodson, S. Castro, B. Page, et al. PaCO2 and alveolar dead space are more relevant than PaO2/FiO2 ratio in monitoring the respiratory response to prone position in ARDS patients: a physiological study. Crit Care, 2011; 15(4): R175.

33. Brun-Buisson, C., C. Minelli, G. Bertolini, L. Brazzi, J. Pimentel, K. Lewandowski, et al. Epidemiology and outcome of acute lung injury in European intensive care units. Results from the ALIVE study. Intensive Care Med, 2004; 30(1): 51-61.

Declarations

Ethics approval and consent to participate

This study was approved by the Institutional Review Board of Taichung Veterans General Hospital Taiwan (IRB number: CE20049B). Given that the research was limited to the secondary use of data previously collected during daily practice and the patients' identity were completely unidentifiable, the informed consent was hence waived

Consent for publication

Not applicable.

Availability of data and material

The datasets consulted during this study are available from the corresponding author on reasonable request.

Competing interests

On behalf of all authors, the corresponding author states that there is no conflict of interest.

Funding

This study was supported by Taichung Veterans General Hospital (TCVGH-1104101C).

Authors' information

[1]Institute of Emergency and Critical Care Medicine, National Yang Ming Chiao Tung University, Taipei, Taiwan.

[2] Department of Critical Care Medicine, Taichung Veterans General Hospital, Taichung, Taiwan.

[3] Division of Chest Medicine, Department of Internal Medicine, Taichung Veterans General Hospital, Taichung, Taiwan.

[4] Division of Pulmonary Medicine, Department of Internal Medicine, Chung Shan Medical University Hospital, Taichung, Taiwan

[5]Department of Thoracic Surgery, Taipei Veterans General Hospital.

[6]Department of Chest Medicine, Taipei Veterans General Hospital

[7]School of Medicine, National Yang Ming Chiao Tung University, Taipei, Taiwan.

[8]School of Post Baccalaureate Medicine, National Chung Hsing University

Authors' contributions

YYY and CMC developed the protocol and designed the study. YYY and OFW collected the data and performed the statistical analysis. YYY and CMC wrote and revised the manuscript. All authors contributed substantially to this work, and all of them revised and have agreed to the final version of the manuscript with full access to all data. All authors read and approved the final manuscript.

Figure Legends

Figure 1. Flow chart of patient enrollment.

Figure 2. Prediction of hospital survival when driving pressure and P/F ratio are combined. DP, Driving pressure; P/F ratio, ratio of partial pressure of oxygen to fraction of inspired oxygen

Table 1. Demographic data of ARDS patients categorized by hospital mortality

Characteristics	All N=370	Survivor N=214 (57.8%)	Non-survivor N=156 (42.2%)	*P* Value [a]
Age (years)	67.7 ± 16.9	68.4 ± 16.6	66.8 ± 17.3	0.313
Male, No. (%)	252 (68.1%)	148 (69.16%)	108 (69.23%)	0.611
BMI (kg/m^2)	23.7 ± 4.9	24.1 ± 4.9	23.2 ± 9.0	0.102
Admission source, No. (%)				
Emergency Room (ER)	303 (81.9%)	177 (82.7%)	126 (80.8%)	0.461
Non-Emergency Room	67(18.1%)	37 (17.3%)	30 (19.2%)	
Type of ICU, No. (%)				
Medical	295 (79.7%)	169 (79.0%)	126 (80.8%)	0.67
Surgical	75 (20.3%)	45 (21.0%)	30 (19.2%)	
Severity scores				
APACHE ll score	28.0 ± 6.9	26.3 ± 6.1	30.4 ± 7.2	<0.0001
SOFA score, Day 1	10.3 ± 3.5	9.5 ± 3.3	11.4 ± 3.6	<0.0001
SOFA score, Day 3	9.3 ± 4.2	8.1 ± 3.4	11.2 ± 4.5	<0.0001
SOFA score, Day 7	7.7 ± 4.2	6.1 ± 3.1	10.4 ± 4.5	<0.0001
Comorbidities, No. (%)				
Cardiovascular disease	102 (27.6%)	63 (29.4%)	39 (25.0%)	0.345
Cerebrovascular disease	75 (20.3%)	48 (22.4%)	27 (17.3%)	0.226
Dementia	26 (7.0%)	17 (7.9%)	9 (5.8%)	0.419
Chronic pulmonary disease	76 (20.5%)	51 (23.8%)	25 (16.0%)	0.066
Rheumatic disease	30 (8.1%)	11 (5.1%)	19 (12.2%)	0.014
Hepatic disease	67 (18.1%)	28 (13.1%)	39 (25.0%)	0.003
Diabetes mellitus	145 (39.2%)	93 (43.5%)	52 (33.3%)	0.049
Renal disease	127 (34.3%)	77 (36.0%)	50 (32.1%)	0.432
Malignancy	141 (38.1%)	56 (26.2%)	85 (54.5%)	<0.0001
Charlson Comorbidity Index (CCI)	3.6 ± 2.9	3.4 ± 2.7	4.1 ± 3.0	0.006
Etiology of ARDS, No. (%)				
Pulmonary, No. (%)	298 (80.5%)	173 (80.8%)	125 (80.1%)	
Pneumonia	255 (68.9%)	153 (71.5%)	102 (65.4%)	0.210
Aspiration	37 (10.0%)	16 (7.5%)	21 (13.5%)	0.058
Pulmonary contusion	6 (1.6%)	4 (1.9%)	2 (1.3%)	0.659
Extrapulmonary, No. (%)	72 (19.5%)	50 (23.4%)	38 (24.4%)	
Sepsis (non-pulmonary source)	48 (13%)	25 (11.7%)	23 (14.7%)	0.387
Trauma or hemorrhagic shock	4 (1.1%)	4 (1.9%)	0 (0%)	0.086
Pancreatitis	4 (1.1%)	1 (0.5%)	3 (1.9%)	0.181
TRALI	10 (2.7%)	7 (3.3%)	3 (1.9%)	0.430
Clinical outcomes				
ICU length of stay	13 (8-22)	15 (9-22)	11 (5-21)	0.002
Hospital length of stay	29.5 (18-46)	34 (24-53)	22 (10-37)	<0.0001

Ventilator-day	13 (7-24)	13 (7-25)	13 (6-23)	0.199

Abbreviations: *ARDS, acute respiratory distress syndrome*; BMI, *Body Mass Index; ICU, intensive care unit;* APCHE II, Acute Physiology And Chronic Health Evaluation II; SOFA, The sequential organ failure assessment score; *CCI, Charlson comorbidity index;* TRALI, transfusion-related acute lung injury; sd , standard deviation; IQR (interquartile range).

[a] *P* value represents comparisons between the survivors and non-survivors ARDS patients.

Table 2 . Mechanical ventilation and arterial blood gas on admission

**Variables**	**All (N=370)**	**Survivors (N=214)**	**Non-survivors (N=156)**	***P* Value [a]**
**Ventilator settings, first day of ARDS(Day0)**				
**Mode, No. (%)**				
Volume -targeted, No. (%)	305 (82.4%)	181 (84.6%)	124 (79.5%)	0.257
Pressure-targeted, No. (%)	65 (17.6%)	33 (15.4%)	32 (20.5%)	
FiO_2	0.6 ± 0.2	0.63 ± 0.22	0.7 ± 0.2	0.022
PaO_2 (mmHg)	103.9 ± 53.3	102.7 ± 52.8	105.6 ± 54.1	0.609
P/F ratio	173.5 ± 88.4	178.6 ± 92.1	166.5 ± 82.8	0.195
PEEP (cmH_2o)	8.8 ± 4.1	8.5 ± 3.2	9.2 ± 5.1	0.117
Tidal Volume (vt/cc/kg)	7.5 ± 1.6	7.6 ± 1.5	7.3 ± 1.7	0.198
RR (1breath/min)	22.2 ± 5.4	21.5 ± 5.2	23.1 ± 5.5	0.004
PIP (mmHg)	24.9 ± 5.9	24.3 ± 5.0	25.7 ± 5.7	0.013
P_{plat} (cmH_2o)	21.5 ± 5.2	20.6 ± 4.4	22.2 ± 6.3	0.012
Driving pressure (cmH_2o)	12.9 ± 4.1	12.3 ± 3.5	13.8 ± 4.8	0.003
Compliance (cmH_2o)	32.9 ± 12.3	34.2 ± 11.7	31.0 ± 12.9	0.023
**Artery blood gas(ABG)-patient in ICU 24hrs**				
pH	7.4 ± 0.1	7.38 ± 0.89	7.33 ± 0.12	0.319
PaO_2 (mmHg)	122.2 ± 98.0	124.6 ± 116.2	119.0 ± 66.0	0.589
$PaCO_2$ (mmHg)	41.8 ± 28.2	42.5 ± 35.0	40.9 ± 14.3	0.575
HCO_3	21.9 ± 5.0	23.6 ± 17.0	22.6 ± 20.1	0.587
**Adjunctive Therapy, No. (%)**				
ECMO	7 (1.9%)	2 (0.9%)	5 (3.2%)	0.113
Lung recruitment maneuver	48 (13.0%)	23 (10.7%)	25 (16.0%)	0.136
Prone position ventilation	23 (6.2%)	9 (4.2%)	14 (9.0%)	0.061
Neuromuscular blockade	159 (43.0%)	75 (35.0%)	84 (53.8%)	<0.0001

Abbreviations: *ARDS, acute respiratory distress syndrome*; RR, respiratory rate; PEEP, positive end-expiratory pressure; FiO_2, inspired fraction of oxygen; PaO_2, partial pressure of oxygen; PaO_2/FIO_2, partial pressure of oxygen to fraction of inspired oxygen; P_{plat}, plateau pressure; VT, tidal volume; PIP, peak inspiratory pressure; ECMO, extracorporeal membrane oxygenation; sd , standard deviation; IQR (interquartile range); ABG, Artery blood gas.

[a] *P* value represents comparisons between the survivors and non-survivors ARDS patients.

Table 3. Multivariate logistic regression model for hospital mortality

Variables	Univariate			Multivariable		
	**Odds ratio**	**95%CI**	***P* Value** [a]	**Odds ratio**	**95%CI**	***P* Value** [b]
Prone						
No		1[Reference]			1[Reference]	
Yes	2.246	(0.946-5.330)	0.067	1.126	(0.350- 3.625)	0.843
Aspiration of gastric contents						
No		1[Reference]			1[Reference]	
Yes	1.925	(0.969- 3.823)	0.061	2.199	(0.878- 5.509)	0.093
Charlson Comorbidity Index (CCI)	1.110	(1.033-1.194)	0.005	1.112	(1.012-1.222)	0.027
MAP, per 1 cmH_2O increment	1.051	(0.995-1.110)	0.077	1.028	(0.957-1.104)	0.449
BMI, per 1 kg/m^2 increment	0.963	(0.923-1.006)	0.092	0.972	(0.915-1.032)	0.356
APACHEII score	1.100	(1.063-1.139)	<0.0001	1.094	(1.045 -1.145)	<0.0001
Driving Pressure, per 1 cmH_2O increment	1.094	(1.033- 1.160)	0.002	1.100	(1.027- 1.179)	0.007

Abbreviations: ARDS, acute respiratory distress syndrome; MAP, Mean Airway Pressure; BMI, Body Mass Index; APCHE II, Acute Physiology And Chronic Health Evaluation II; CCI, Charlson comorbidity index; CI., confidence interval.

[a] Univariate analysis.

[b] All variables included in the multivariable analysis are reported in this Table

Click here to access/download;Figure;Figure 1 flow chart of patient enrollment.jpg

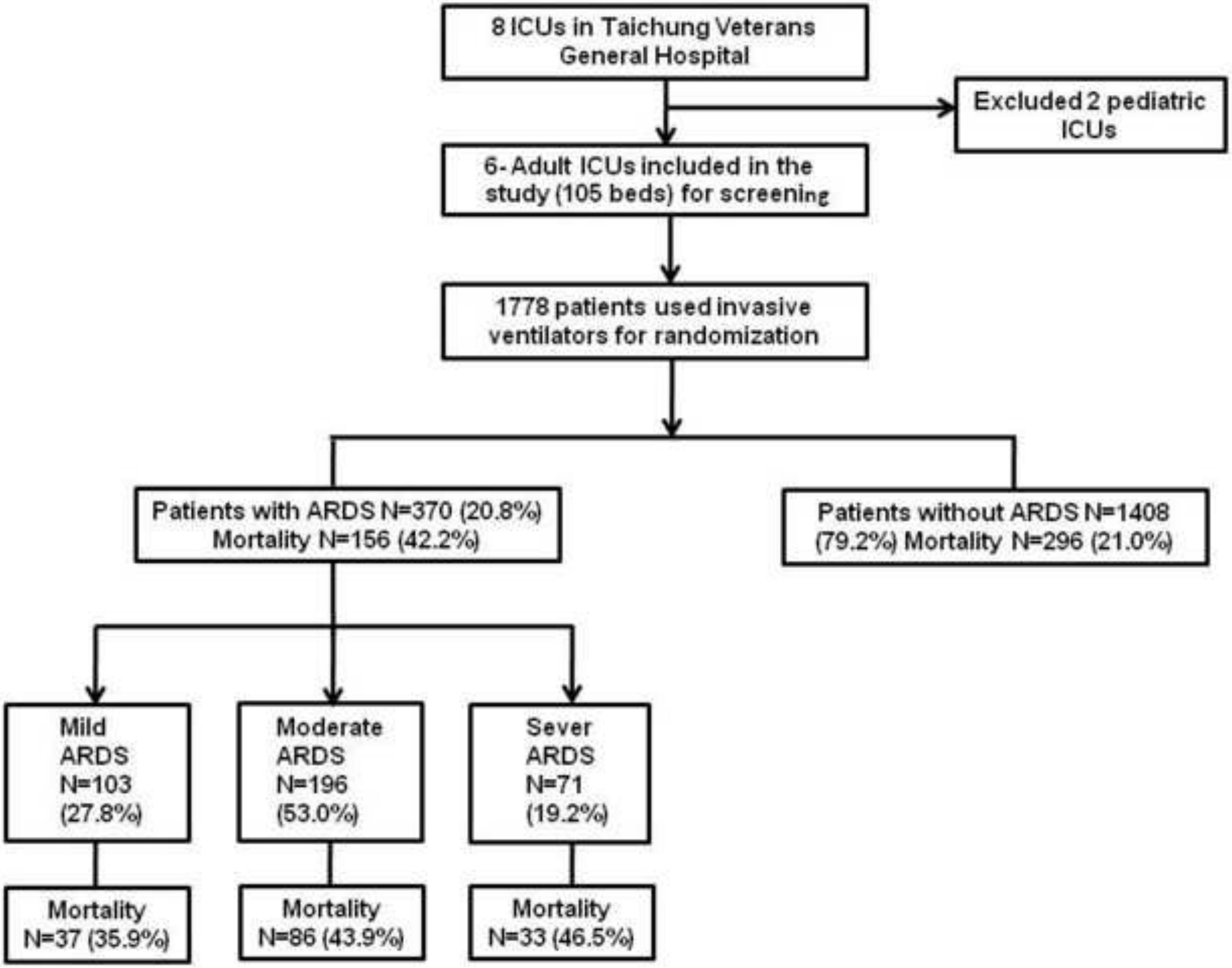

Figure2

Click here to access/download;Figure;Figure 2 Prediction of hospital survival.jpg

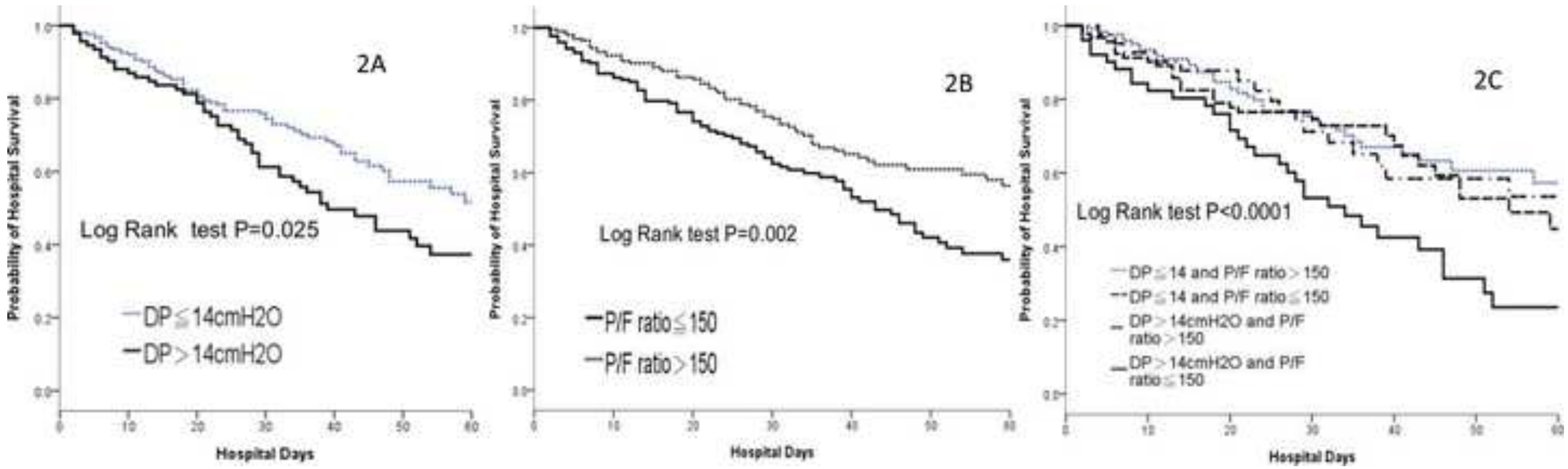

Click here to access/download

Supporting Information

Table S1 Characteristics of ARDS patients (severity).docx

Click here to access/download
Supporting Information
Table S2 Mechanical ventilation by ARDS (severity).docx

Click here to access/download
Supporting Information
Table S3Characteristics of ARDS patients (DP).docx

Click here to access/download
Supporting Information
Table S4Mechanical ventilation (DP).docx
