# Supplementary material for: A combination of oxygenation and driving pressure can provide valuable information in predicting the risk of mortality in ARDS patients
Source: PLoS One. 2023 Dec 13;18(12):e0295261. doi: 10.1371/journal.pone.0295261 (PMC10718417; doi:10.1371/journal.pone.0295261)
Supplement: Attachment — Submitted filename: Responses to Reviewers (PONE-D-23-11371R1)2nd20231103.docx [file pone.0295261.s008.docx]

**Responses to Reviewers (PONE-D-23-11371R1)**

**Responses to the Reviewers**

We would like to express our gratitude to the reviewers for their thorough evaluation of our manuscript and for providing valuable comments and suggestions. We have carefully addressed all of their remarks, as detailed below, and provided our responses to specific comments. The revised sections are highlighted in red font. We believe that, as a result of the reviewers' recommendations, our manuscript has become clearer, and we hope that these changes will be deemed satisfactory.

Reviewer #1: I would like to thank the authors for revising the manuscript. You adequately addressed most of my comments.

**Responses to Reviewer #1**

**Comment 1-1:**

1- However, it seems that I didn’t make my first previous comment clear. I was not commenting on mortality of different ARDS severities. I was commenting on independent predictors of mortality in ARDS in general, which is the primary outcome of the study.
   So, when you say in the abstract:
   Page 5 & 6 (lines 85 – 87): Both oxygenation and driving pressure were independently associated with mortality in ARDS (p=0.014).
   You give the impression that each one separately is a predictor of mortality, which is not the case. According to table S5: It is the combined driving pressure and P/F ratio.
   So, please make it clear: driving pressure > 14 and P/F ratio ≤ 150 was an independent predictor of mortality (OR = 2.497(95% CI: 1.201-5.191); p = 0.014).

**Response:**

We appreciate the reviewer for reminding us of this important issue. The statement in abstract section is revised as following: In a multivariate logistic regression model, combination of driving pressure (DP) > 14cmH_2_O and oxygenation (P/F ratio) ≤150 was an independent predictor of mortality (OR 2.497, 95% CI 1.201-5.191, *p*=0.014). (Page 5, lines 82 – 85)

**Comment 1-2:**

2- Table S5 is the same as table 3 in the manuscript, with the addition of 4 categories of different combinations of driving pressure and P/F ratio, so why not simply add those categories to table 2.

**Response:**

We thank the reviewer for this valuable suggestion. We have revised Table 2 and Table 3. According to the reviewer`s suggestion, we also delete Table S5, as it is similar to Table 3, and added 4 categories. We provide a statement to clarify this issue. The manuscript is also revised as following:

1. In the Abstract section: In a multivariate logistic regression model, combination of driving pressure (DP) > 14cmH_2_O and oxygenation (P/F ratio) ≤150 was an independent predictor of mortality (OR 2.497, 95% CI 1.201-5.191, *p*=0.014).(page 5, line 82-85)
2. In the Result section:
(1) We further divided the patients into four groups by DP and oxygenation. Patients with DP>14 and P/F ratio≤150 had the highest mortality rate (31/52= 60.8%) (Table 2). (page 14, line 226-228)
(2) A multivariate regression model was adopted to adjusting for possible confounders, including prone position ventilation, aspiration of gastric content, CCI, RR, FiO_2_, mean arterial pressure, BMI, APACHE II score and DP and P/F ratio combination groups. (page 16, line 258-261)
(3) Two independent factors were identified, including APACHE II score (adjusted odds ratio (OR) 1.089, 95% confidence interval (CI) 1.045-1.135; p<0.0001) and combination of DP/oxygenation (DP>14/P/F ratio≤150, OR 2.497, 95% CI 1.201-5.191, p=0.014) (Table 3). (page 16, line 262-266)
(4) Additionally, our study identified combination of oxygen and respiratory mechanics, specifically in DP>14 and P/F ratio≤150, as an independent risk factor (Table 3, p=0.014). (page 16, line 269-271)
3. In the Discussion section: We found APACHE II scores and a combination of oxygenation and driving pressure were independent mortality risk factors. A combination of oxygenation status and respiratory mechanics gives better outcome prediction in ARDS patients. (page 18, line 282-285)
4. In the Conclusion section: ARDS is common among critically ill patients who are admitted to the ICU and who use invasive mechanical ventilation. Hospital mortality remains high and could potentially be improved by adherence to currently available evidence. Combining oxygenation status and respiratory mechanics may better predict ARDS patients at risk of mortality. (page 23, line 373-377)
5. **Comment 1-3:**

3- Page 17, lines 274-277: You still refer to results of Kaplan Meier curves and Log rank tests as (Mortality rates). It is Survival.

**Response:**

We would like to thank the reviewer for the suggestion to our manuscript. In response, we revised the statement in the Result section as following: Patients with a driving pressure >14 cmH_2_O (Fig 2A, *p*=0.025) and a P/F ratio ≤150 (Fig2B, *p*=0.002) had a lower hospital survival rate. By combining these factors, patients with worse oxygenation and higher driving pressure had the lowest hospital survival rate (Fig 2C, *p*<0.0001). (page 17, line 274-277)

4- In the conclusion (page 23, lines 375, 376) you stated that CCI is an independent predictor of outcome, when it isn’t: OR = 1.072[95% CI: (0.985-1.166)]; p = 0.106
I think that your conclusion should be: Driving pressure alone, or in combination with P/F ratio are independent predictors of mortality.

**Response:**

We thank the reviewer for bringing this detail to our attention. In response to the reviewer’s suggestion, we have revised the statement in the Conclusion section as follows:

ARDS is common among critically ill patients who are admitted to the ICU and who use invasive mechanical ventilation. Hospital mortality remains high and could potentially be improved by adherence to currently available evidence. Combining oxygenation status and respiratory mechanics may better predict ARDS patients at risk of mortality. (page 23, line 373-377)

**Responses to Journal Requirements (PONE-D-23-11371R1)**

Journal Requirements:

Please review your reference list to ensure that it is complete and correct. If you have cited papers that have been retracted, please include the rationale for doing so in the manuscript text, or remove these references and replace them with relevant current references. Any changes to the reference list should be mentioned in the rebuttal letter that accompanies your revised manuscript. If you need to cite a retracted article, indicate the article’s retracted status in the References list and also include a citation and full reference for the retraction notice.

**Response:**

**Removed references:**

We remove these references as per the previous reviewer`s suggestion (PONE-D-23-11371) to stay on topic and avoid discussing unrelated issues. Therefore, we have removed all content related to DNR from the article, including the reference citation.

**22.** Phua, J., G.M. Joynt, M. Nishimura, Y. Deng, S.N. Myatra, Y.H. Chan, et al. Withholding and withdrawal of life-sustaining treatments in low-middle-income versus high-income Asian countries and regions. Intensive Care Med, 2016; 42(7): 1118-27.

**23.** Lautrette, A., M. Garrouste-Orgeas, P.M. Bertrand, D. Goldgran-Toledano, S. Jamali, V. Laurent, et al. Respective impact of no escalation of treatment, withholding and withdrawal of life-sustaining treatment on ICU patients' prognosis: a multicenter study of the Outcomerea Research Group. Intensive Care Med, 2015; 41(10): 1763-72.

**Added references:**

We included these references for the reviewer`s suggestions to cite within the first 7 lines of the Introduction section of the manuscript.

1. Thompson, B.T., R.C. Chambers, and K.D. Liu. Acute Respiratory Distress Syndrome.NEnglJMed,2017;377(6):562-572.https://doi.org/10.1056/NEJMra1608077 PMID:28792873.

2. Ware, L.B. and M.A. Matthay. The acute respiratory distress syndrome. NEnglJMed,2000;342(18):1334-49.https://doi.org/10.1056/nejm200005043421806 PMID:10793167.

3. Cardinal-Fernández, P., J.A. Lorente, A. Ballén-Barragán, and G. Matute-Bello. Acute Respiratory Distress Syndrome and Diffuse Alveolar Damage. New Insights on a Complex Relationship. AnnAm Thorac Soc, 2017; 14(6):844-850.https://doi.org/10.1513/AnnalsATS.201609-728PS PMID:28570160.

4. Matthay, M.A., R.L. Zemans, G.A. Zimmerman, Y.M. Arabi, J.R. Beitler, A. Mercat, et al. Acute respiratory distress syndrome. Nat Rev Dis Primers, 2019; 5(1): 18. https://doi.org/10.1038/s41572-019-0069-0 PMID:30872586.

5. Sweeney, R.M. and D.F. McAuley. Acute respiratory distress syndrome. Lancet,2016;388(10058):2416-2430.https://doi.org/10.1016/s0140-6736(16)00578-x PMID:27133972.
